# Electrochemical DNA-Sensor Based on Macrocyclic Dendrimers with Terminal Amino Groups and Carbon Nanomaterials

**DOI:** 10.3390/s23104761

**Published:** 2023-05-15

**Authors:** Tatjana Kulikova, Rezeda Shamagsumova, Alexey Rogov, Ivan Stoikov, Pavel Padnya, Igor Shiabiev, Gennady Evtugyn

**Affiliations:** 1 A.M. Butlerov’ Chemistry Institute, Kazan Federal University, 18 Kremlevskaya Street, 420008 Kazan, Russia; wefy2009@yandex.ru (T.K.); rezeda84190@mail.ru (R.S.); ivan.stoikov@mail.ru (I.S.); pavel.padnja@kpfu.ru (P.P.); shiabiev.ig@yandex.ru (I.S.); 2 Interdisciplinary Center of Analytical Microscopy, Kazan Federal University, 18 Kremlevskaya Street, 420008 Kazan, Russia; 1arogov@kpfu.ru; 3 Analytical Chemistry Department, Chemical Technology Institute, Ural Federal University, 19 Mira Street, 620002 Ekaterinburg, Russia

**Keywords:** thiacalix[4]arene, DNA sensing, supramolecular dendrimers, cyclic voltammetry, electrochemical impedance spectroscopy, doxorubicin

## Abstract

The assembling of thiacalix[4]arene-based dendrimers in *cone*, *partial cone*, and *1,3-alternate* configuration on the surface of a glassy carbon electrode coated with carbon black or multiwalled carbon nanotubes has been characterized using cyclic voltammetry, electrochemical impedance spectroscopy, and scanning electron microscopy. Native and damaged DNA were electrostatically accumulated on the modifier layer. The influence of the charge of the redox indicator and of the macrocycle/DNA ratio was quantified and the roles of the electrostatic interactions and of the diffusional transfer of the redox indicator to the electrode interface indicator access were established. The developed DNA sensors were tested on discrimination of native, thermally denatured, and chemically damaged DNA and on the determination of doxorubicin as the model intercalator. The limit of detection of doxorubicin established for the biosensor based on multi-walled carbon nanotubes was equal to 1.0 pM with recovery from spiked human serum of 105–120%. After further optimization of the assembling directed towards the stabilization of the signal, the developed DNA sensors can find application in the preliminary screening of antitumor drugs and thermal damage of DNA. They can also be applied for testing potential drug/DNA nanocontainers as future delivery systems.

## 1. Introduction

Dendrimers are nanosized radially symmetric molecules with a well-defined monodisperse structure consisting of tree-like arms [1]. Symmetric branching units are built around a small core, and their terminal functional groups can be functionalized to modify the charge, the hydrophobicity of the molecule, and the steric arrangement of the dendrimer core. Dendrimers have found increasing application as nanocontainers for targeted drug delivery, such as specific sorbents, DNA vectors, and components of medications [2,3,4,5]. In biosensor design, dendrimers have been utilized as carriers for the deposition of metal nanoparticles [6] and the immobilization of aptamers and antibodies [7,8,9]. They offer a higher number of binding sites associated with terminal functional groups over common carriers and can protect biomolecules by preventing their denaturation caused by organic solvents and other damaging factors. The combination of dendrimers with conductive materials including graphene materials [10,11], carbon nanotubes [12,13,14], carbon nitride [15], and carbon black [16] makes it possible to overcome the main drawback of the dendrimers in the assembly of sensors, i.e., their low electroconductivity and lack of redox activity.

Among other targets, DNA–dendrimer interactions have attracted growing interest during the past decade due to the importance of the goals related to the design of new DNA/RNA vaccines [17], oligonucleotide-based drugs [18], antitumor drug delivery, and transfection systems [19]. Meanwhile, the development of the DNA/RNA drugs requires further developing the protection and storage systems to prevent their enzymatic digestion and to provide their delivery and release in the target tissues [20].

Implementation of the DNA components in a supramolecular complex via multiple noncovalent interactions is one of the promising approaches in the design of such systems [21]. The complexation mentioned can be based on polyelectrolytes [22,23], solid lipid nanoparticles, liposomes, and vesicles [24,25,26].

Macrocyclic compounds, e.g., cyclodextrins [27], pillararenes [28,29,30], and thiacalixarenes [31,32], offer new opportunities in the design of DNA complexes due to their high selectivity of recognition and binding, the variety of functional groups participating in the target interactions, their variable solubility in water, and their accessibility for biological targets. Phosphate groups of the DNA backbone can be involved in binding counter ions, including positively charged terminal groups of the macrocycle substituents. Moreover, the formation of supramolecular complexes can alter the interaction of the biopolymer with small molecules, e.g., antitumor drugs. Thus, doxorubicin showed higher activity in the DNA intercalation in the presence of β-cyclodextrin [33].

Recently, a number of examples of the DNA–macrocycle interactions have been utilized in electrochemical sensors based on a calixarene and pillararene platform with charged or lipophilic substituents [34,35,36]. They showed remarkable dependence on the aggregation of appropriate counterparts on the structure and spatial arrangement of the functional groups in the macrocyclic ligands both in organic solvents and on the electrode interface. The electrochemical properties of the electrodes modified with the DNA–macrocycle complex were applied for the characterization of the complexation and assessment of the DNA-specific interactions with small ions. 

In this work, we describe the use of cationic dendrimers on a thiacalix[4]arene platform in the DNA sensor and discuss the influence of the macrocyclic core configuration and the underlaying modifier on the DNA implementation and the DNA–drug interaction detected by electrochemical approaches.

## 2. Materials and Methods

### 2.1. Reagents

**Figure 1:**
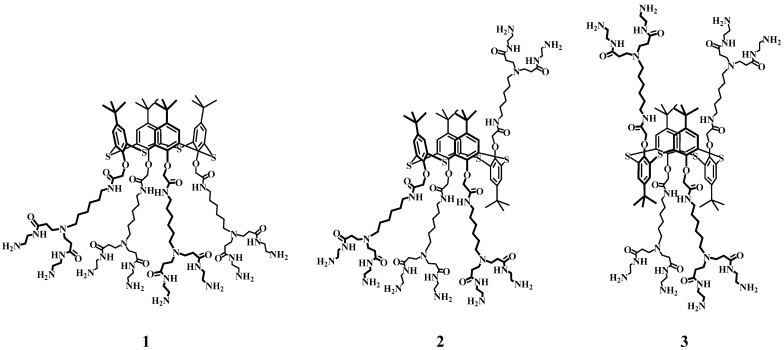
Chemical structure of the substituted thiacalix[4]arenes in configuration *cone* (**1**), *partial cone* (**2**), and *1,3-alternate* (**3**).

5,11,17,23-Tetra-*tert*-butyl-25,26,27,28-tetrakis[N-(6-(*N,N*-di(N-(2-aminoethyl)carba- moylethyl)amino)hexyl)carbamoylmethoxy]-2,8,14,20- tetrathiacalix[4]arenes **1**–**3** (Figure 1) bearing eight terminal amino groups in *cone*, *partial cone*, and *1,3-alternate* configurations were synthesized at the Organic and Medicinal Chemistry Department of Kazan Federal University as described elsewhere [37].

The structure and purity of the compounds were proved by ^1^H NMR and ^13^C NMR spectroscopy (Bruker Avance 400 spectrometer (Bruker, Mannheim, Germany), MALDI mass-spectrometry (Ultraflex III mass spectrometer, Perkin Elmer, Waltham, MA, USA), FTIR ATR spectroscopy (Spectrum 400 IR spectrometer, Perkin Elmer, Waltham, MA, USA), and elemental analysis (Perkin–Elmer 2400 Series II instrument, spectrometer, Perkin Elmer, Waltham, MA, USA). The configurations of the thiacalix[4]arene **1**–**3** were quite stable in the working conditions and could not transform into each other because of the bulky tert-butyl substituents at the upper rim of the thiacalix[4]arene core that prevent free rotation around the bridging sulfur atoms.

DNA from salmon testes (Cat. No D1626, A_260/280_ = 1.4), doxorubicin hydrochloride (Cat. No D1515, 98–102%), idarubicin hydrochloride (Cat. No I1656, >98%), daunorubicin hydrochloride (Cat. No 30450, >90%), potassium hexacyanoferrate (III) (99%), potassium hexacyanoferrate (II) (98.5–102%), Methylene green zinc chloride salt (>80%), hydroquinone, multiwalled carbon nanotubes (MWCNTs), and functionalized carboxylic acid (Cat. No 755125) were purchased from Sigma-Aldrich, Dortmund, Germany; DNA from chicken erythrocytes (Cat. No 04021, average mol. mass 1.2 MDa, A_260/280_ = 1.9) was purchased from “Reanal”, Budapest, Hungary; chitosan (mol. weight 100,000–30,000 D) was purchased from Acros Organics, Geel, Belgium; and carbon black (CB, N220, >99.95% C) was purchased from Imerys, Willebroek, Belgium. Doxorubicin—TEVA^®^ and Doxorubicin-LANS^®^ were purchased in the local pharmacy market. Working solutions were prepared with Millipore Q^®^ water (Simplicity^®^ water purification system, Merck-Millipore, Molsheim, France). All other reagents were of analytical grade. Electrochemical measurements were performed in 0.1 M HEPES.

### 2.2. Apparatus

Glassy carbon electrodes (GCEs) modified with carbon materials and dendrimer–DNA complexes were characterized using direct current (DC) voltammetry at ambient temperature with the Autolab PGSTAT 302N (Metrohm Autolab b.v., Utrecht, the Netherlands). Voltammetric measurements were performed in the 5 mL three-electrode cell equipped with GCE (ALS Co., Ltd., Tokyo, Japan, Cat. No 012744, geometric area 0.283 cm^2^) and modified with the above components as a working electrode, with Pt wire (ALS Co., Ltd., Cat. No 002233) as the auxiliary electrode, and the Ag/AgCl/3 M KCl (Metrohm Autolab Cat. No 6.0726.107) reference electrode. Electrochemical impedance spectroscopy (EIS) measurements were performed with the Autolab PGSTAT 302N equipped with the FRA2 module (frequency range 100 kHz–0.04 Hz, sine potential 5 mV). Equilibrium potential was assessed as a half-sum of the peak potentials recorded in the equimolar mixture of 10 mM [Fe(CN)_6_]^3−/4−^ ions. The EIS parameters were calculated from the Nyquist diagram corresponding to the *R(RC)(RC)* equivalent circuit using the NOVA 1.11 software (Metrohm Autolab). 

Scanning electron microscopy (SEM) images of the electrode coatings were obtained with the high-resolution field emission scanning electron microscope Merlin™ (Carl Zeiss, Jena, Germany).

### 2.3. GCE Preliminary Modification and Sensor Assembling

**Figure 2:**
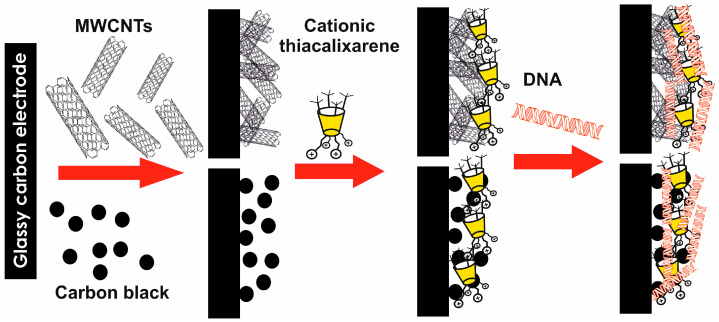
The outline of the DNA sensor assembling.

The GCE was mechanically polished with an alumina polishing kit and washed with acetone and deionized water. After that, it was immersed together with the reference and auxiliary electrodes in the working buffer and the potential was cycled between −1.0 and 1.0 V in 0.1 M H_2_SO_4_ until stabilization of the background current. Then, the electrode was washed again. The CB or MWCNTs suspension was prepared by sonication of the CB (MWCNTs) in chitosan (1.35 mg in 1 mL of 0.375% chitosan dissolved in 0.05 M HCl). For the electrode modification, 2 μL of the CB (MWCNTs) suspension was mixed with 1 μL of 1.0 M NaOH, drop casted on the electrode, and dried at 50 °C for 20 min. Then, the GCE was covered with 5 μL of the thiacalix[4]arene **1**–**3** solution (concentration ranged from 5.0 μM to 0.5 mM) separately or together with 0.4 mg/mL DNA from salmon testes. In some experiments, the DNA concentration was varied or the DNA was thermally denatured prior to drop casting to the electrode surface by slow heating to 90 °C for 30 min. followed by a sharp cooling in the ice bath. Oxidation of DNA was performed by mixing its solution with the CuSO_4_/H_2_O_2_ mixture for 10–60 min. Final concentrations of the reagents mentioned below refer to the mixtures/solutions prepared. The assembling of the DNA sensors is outlined in Figure 2.

## 3. Results

As was previously shown [38], DNA from salmon testes can bind thiacalix[4]arene **1** in a *cone* configuration with the formation of the complexes leaving access to small molecules in the DNA helix. The possibility of the transfer of the macrocycle on the GCE’s surface covered with CB was confirmed using SEM and EIS data. In this work, we compared the complexation of various configurations of the macrocycle with the same number of terminal amino groups after their deposition on the carbon materials as supports for the complex assembling (CB vs. MWCNTs). The properties of the complexes formed, i.e., their permeability for redox indicators of different charges, were assessed through comparison of the redox peaks of ferricyanide ion, Methylene green, and hydroquinone recorded in the DC mode. The choice of the redox indicators is explained by the significant role of electrostatic interactions in the performance of appropriate DNA sensors consisting of macrocycles and DNA molecules. Indeed, phosphate residues of the DNA backbone control the diffusional transfer of an anionic [Fe(CN)_6_]^3−^ ion, positively charged Methylene green, and neutral hydroquinone molecules differently. Their comparison as redox indicators made it possible to distinguish the electrostatic and diffusional limitations of the reactions taking place on the electrode interface. All the voltammograms recorded in the redox indicator solutions were stabilized to the fifth cycle of the potential. In the following discussion, stabilized voltammograms are considered.

**Figure 3:**
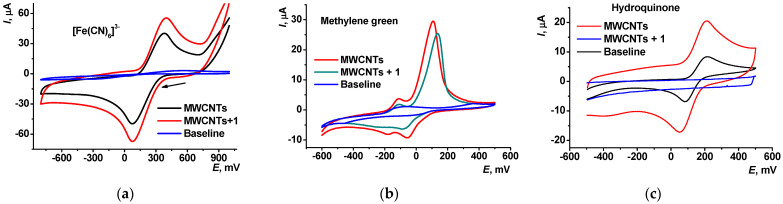
Cyclic voltammograms recorded in 0.1 M HEPES, pH = 7.0, in the presence of 5 mM [Fe(CN)_6_]^3−^ (**a**), 0.5 mM Methylene green (**b**), and 0.5 mM hydroquinone (**c**). GCE modified with MWCNTs and MWCNTs–thiacalixarene **1**, scan rate 100 mV/s. Baseline corresponds to the voltammogram recorded without redox indicator in the same buffer. Arrow indicates the direction of the potential canning.

The deposition of the DNA–thiacalix[4]arene dendrimers (see chemical structures on Figure 1 and the assembling protocol in Figure 2) did not significantly alter the shape and position of the reversible peaks of the redox indicators on cyclic voltammograms. As an example, Figure 3 represents the cyclic voltammograms recorded on the electrodes covered with the MWCNTs prior to and after the deposition of 0.5 mM thiacalix[4]arene **1** (aliquot 2 μL per electrode). Similar results obtained on the CB support were provided in [38].

In the case of ferricyanide ions and hydroquinone, the symmetrical pair of peaks changed their height after the macrocycle deposition within the same potential region. Methylene green was able to adsorb on the electrode so that its anodic peak was higher and sharper than the corresponding cathodic peak on the reversed scan. A small reversible peak pair at lower potentials (−180 and −107 mV) corresponded to the redox conversion of the adsorbed molecules of the redox indicator. It remained visible on the first scan recorded after transfer of the modified electrode into the fresh buffer with no redox indicator but decreased down to full disappearance within four–five following scans of the potential. This indicates the reversible character of the Methylene green adsorption. This behavior coincides well with previously described Methylene green redox conversion on the MWCNT-modified electrode [39]. 

Deposition of the MWCNTs and CB on bare GCE improved reproducibility of the redox signals and extended the lifetime of the sensors. Peak currents of the redox indicators increased against the bare electrode due to a higher effective working area caused by the carbon nanomaterials deposited. This was confirmed by comparison of the peaks recorded in the 10 mM [Fe(CN)_6_]^3−^ solution using the Randles–Ševčik equation. Loading of 2 μL of the suspensions per electrode resulted in an increase in the reduction peak current by 1.9 times for the CB and 2.0 for the MWCNTs (with chitosan as a film-forming component of the surface layer). The quantities of the carbon materials loaded were selected as a compromise between the requirements of a full surface coverage and of the mechanical stability of the layer obtained. For smaller amounts of modifiers, the gapes in the surface layer affected the electron exchange assessment and reproducibility of the voltammetric and impedimetric responses. Higher quantities of carbon materials resulted in the instability of the layer and the partial removal of the aggregates during the operation with the sensor, i.e., its washing, transfer to other solutions, stirring of the working solution, etc.

### 3.1. Voltammetric Characterization of the DNA–Thiacalix[4]arene Complexes

#### 3.1.1. The Influence of the Macrocycle Configuration

**Figure 4:**
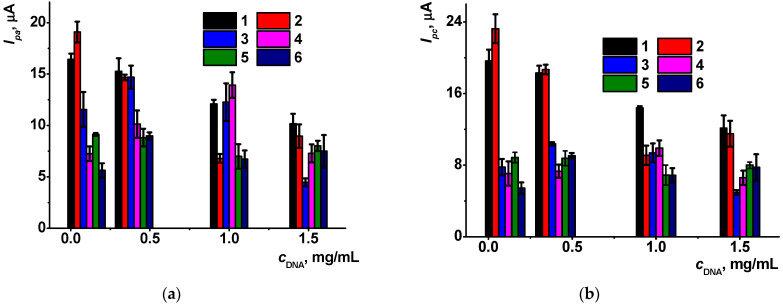
Anodic (**a**) and cathodic (**b**) peak potential currents on voltammograms of ferricyanide (1, 2), Methylene green (3, 4), and hydroquinone (5, 6) measured on GCE/CB electrode covered with thiacalix[4]arene **1** and various amounts of native (1, 3, 5) and denatured (2, 4, 6) DNA. Redox indicator concentration 0.1 mM, measurements in HEPES, pH = 7.0, scan rate 100 mV/s. Average ± S.D. for three individual sensors.

Figure 4 represents the changes in the peak currents recorded on the GCE covered with the CB and thiacalix[4]arene **1** in the presence of native and thermally denatured DNA. Implementation of the DNA by its physical adsorption onto the CB/thiacalix[4]arene layer mostly decreased the peak currents because of the entrapment of nonconductive and redox inactive biopolymer in the surface layer. The only exception was obtained for low amounts of the deposited DNA (0.1 and 0.4 mg/mL, 2 μL aliquot). We assumed the interaction with terminal amino groups of the macrocycle compensated for the negative charge of the phosphate residues in the DNA helix so that the diffusional limitations of the indicator access to the electrode became insignificant. The signals of Methylene green increased with the DNA quantities probably due to electrostatic accumulation of the dye on the DNA molecules. This redox indicator is unable to intercalate the DNA helix because of the nitro group at the phenothiazine core of the molecule that distorted the planar structure required for this process. Methylene green is accumulated at the minor grooves of the DNA retaining its electrochemical activity both in its free and bonded state. The mechanism of interaction of the DNA with Methylene green has been proved using spectrophotometric and electrochemical methods [40].

Hydroquinone peaks were less sensitive to the DNA implementation and did not show alteration with the DNA denaturation.

**Figure 5:**
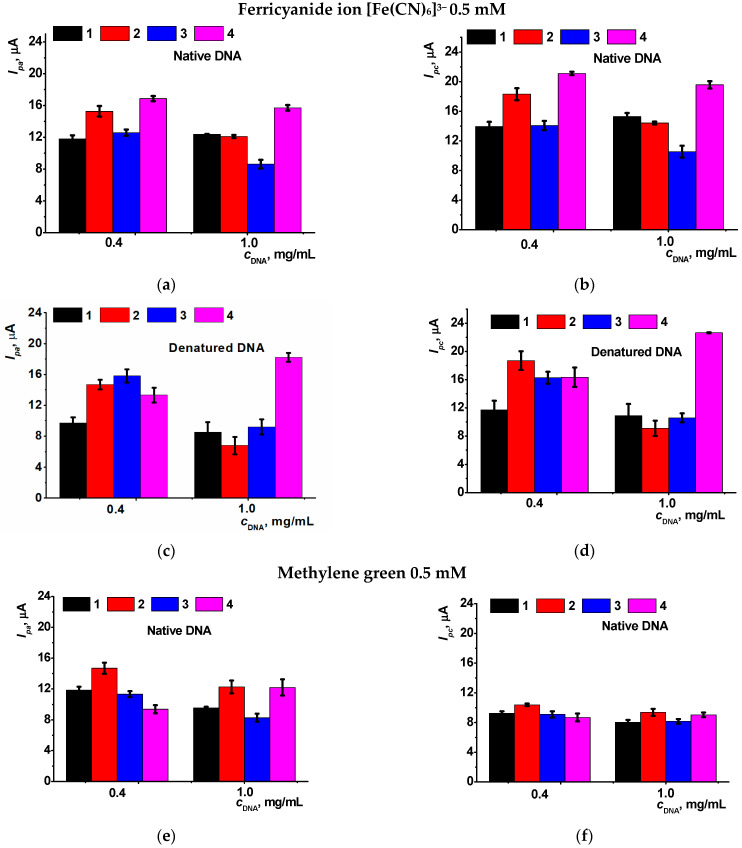

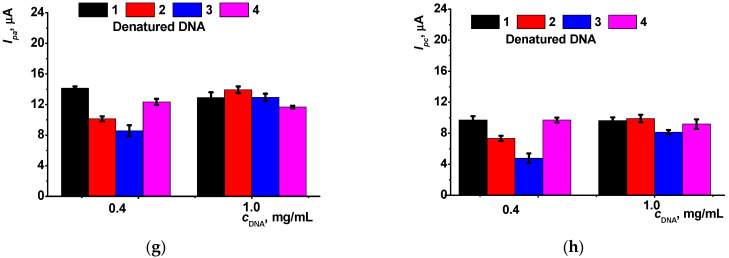
Anodic (**a**,**c**,**e**,**g**) and cathodic (**b**,**d**,**f**,**h**) peak currents of redox indicators measured on the GCE/CB electrode covered in the absence (1) and in the presence of thiacalix[4]arenes **1** (2), **2** (3), and **3** (4) in the presence of native and denatured DNA. Redox indicator concentration 0.5 mM, measurements in HEPES, pH = 7.0, scan rate 100 mV/s. Average ± S.D. for three individual sensors.

Similar experiments were performed with thiacalix[4]arenes **2** (*partial cone*) and **3** (*1,3-alternate*). Hydroquinone was excluded from consideration because of the lower DNA influence on its signal. The comparison of the peak currents obtained for 0.4 and 1.0 mg/mL DNA and 50 μM thiacalixarenes in the mixture is presented in Figure 5.

For the ferricyanide indicator (Figure 5a–d), *partial cone* **2** showed minimal influence of the DNA loading, both native and thermally denatured, on the peak currents. 

For *partial cone*, spatial coordination of the terminal amino groups did not provide full binding in the complex. Even bonded with the biopolymer, the macrocycle left two amino groups on the opposite side of the macrocycle core plane facing the solution. This resulted in compensation of higher resistance assumed for biopolymer inclusion by electrostatic attraction with the anionic ferricyanide indicator. In the case of the *1,3 alternate* **3**, electrostatic interactions prevailed over the diffusional limitations so that the peaks became higher than those of the macrocycles deposited on the electrode alone without DNA molecules (columns 1 and 4 in Figure 5). Thermal treatment of the DNA prior to its deposition on the electrode partially destroyed the coordination of the charged groups of counterparts so that electrostatic attraction became a dominating factor for all the configurations of the macrocycles studied. At higher DNA concentration (1.0 mg/mL), the symmetry of the macrocyclic core became more important for the ferricyanide access and the *1,3-alternate* that showed maximal redox peaks on appropriate voltammograms (column 4 in Figure 5c,d).

**Figure 6:**
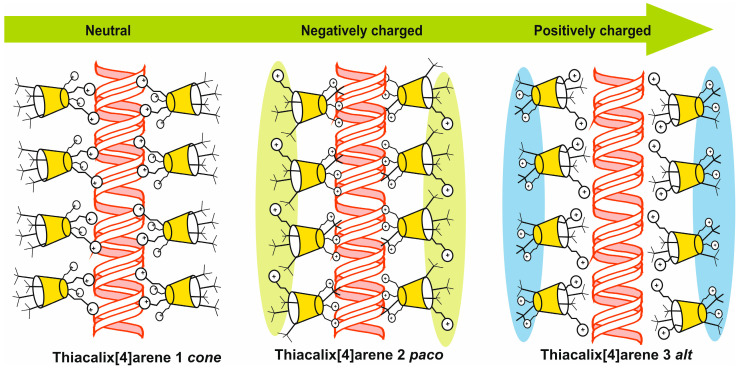
The interaction of DNA with various configurations of the macrocycles.

The influence of the macrocycle configuration on the DNA binding is illustrated in Figure 6. Previously, the formation of similar aggregates with the PAMAM–calixarene dendrimers and DNA in polar organic solvents was proved by UV-Vis spectroscopy, dynamic light scattering, and transmission electron microscopy [37]. Taking into account the charge distribution and the number of cationic centers in the macrocycle, it can be concluded that thiacalix[4]arene **1** exerts a maximal shielding effect on the phosphate residue’s charge, whereas *partial cone* **2** conformer retains the negative charge of the associates reduced against the free DNA, and *1,3-alternate* **3** results in a the formation of a recharged (cationic) product. This agrees with the different behavior of the appropriate redox indicators tested. The influence of native DNA was quite stable within incubation period that varied from 10 to 60 min. For thermally denatured DNA, the signal ferricyanide ions remained the same but the Methylene green accumulated rather slowly so that its peak currents increased by 100–160% of the initial value. Additional treatment of the CB/thiacalix[4]arene/DNA layer with glutaraldehyde did not alter the signal of redox indicators so that possible reversibility of DNA implementation can be excluded from consideration.

#### 3.1.2. The Influence of the Support

**Figure 7:**
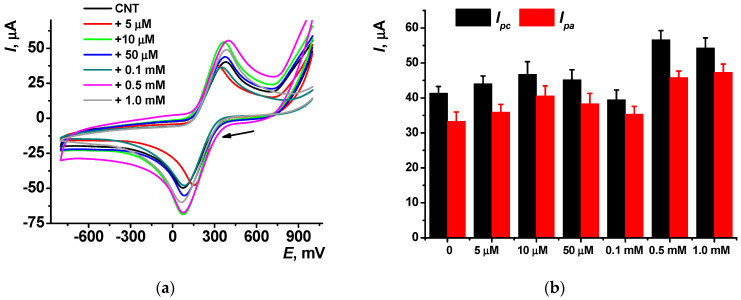
(**a**) Cyclic voltammograms of 5.0 mM K_3_[Fe(CN)_6_] recorded on the GCE covered with MWCNTs (2 μL of 1.3 mg/mL suspension in 0.375% chitosan) and thiacalix[4]arene **1** (5–1000 μM in 2 μL aliquot per electrode). Arrow indicates the direction of the potential scanning. (**b**) The comparison of the reduction and oxidation peak currents. Average ± S.D. for four individual sensors.

To estimate the possible influence of the carbon nanomaterials deposited on the GCE prior to the macrocycle/DNA deposition, thiacalix[4]arene **1** was chosen because it showed a more flexible signal after contact with the DNA (see Figure 4). Figure 7 shows voltammograms recorded in 5.0 mM [Fe(CN)_6_]^3−^ solution after deposition of increasing quantities of thiacalix[4]arene **1** on the electrode.

Increased loading of the macrocycle resulted in a higher peak potential difference attributed to a slower electron exchange caused by implementation of the charged but nonconductive molecules on the electrode interface. Meanwhile, the equilibrium potential assessed as a half-sum of the anodic and cathodic peak potentials remained constant. Probably, electrostatic attraction of the redox indicator molecules charged oppositely to the terminal groups of the macrocycle compensated for the higher diffusional hindrance of their diffusional transfer to the electrode. This is indirectly confirmed by an increase in appropriate peak currents at low macrocycle loading (5–50 μM). At higher macrocycle loading, changes in the ferricyanide peak currents became irregular due to the possible aggregation of the macrocycle molecules and random alternation of its effective charge on the electrode interface.

Similar experiments were performed by varying the DNA concentration for a constant content of the macrocycle **1** (Figure 7). Increasing DNA concentration changed the [Fe(CN)_6_]^3−^ peak currents depending on the thiacalixarene content. At high macrocycle deposition (0.5 mM), low DNA quantities increased the peaks recorded until the DNA concentration reached 0.4 mg/mL. At higher DNA quantities, the peaks of the redox indicator were stabilized and then started slowly decreasing. At minimal thiacalix[4]arene loading (0.005 mM), the peaks of the redox indicator were regularly decreased with increasing DNA concentration within the whole range of its variation. At middle thiacalix[4]arene level (0.05 mM), changes of the [Fe(CN)_6_]^3−^ peak currents were rather small and irregular.

We suppose the observed phenomena are related to a different role of the DNA molecules in the surface layer. They can promote the access of the anionic redox indicator by preferable coordination of the macrocyclic dendrimer followed by aggregation of the film and formation of through channels to the GCE surface. At higher concentration of thiacalix[4]arene, the appropriate reaction resulted in both shielding the negative charge of the DNA phosphate residues and in making the nonconductive layer on the electrode thicker. Both trends affect the peak currents observed in the opposite directions so that different surface concentrations of the reactants showed different behavior in DC mode of the signal measurement.

### 3.2. SEM Measurements

It should be noted that nominal concentrations of the solutions drop casted on the electrode were mentioned in the above description. Indeed, the real ratio of the reagents depends both on the self-assembling and the aggregation of the reactants. Excessive amounts of both DNA and macrocycle could leave the electrode surface during the washing step.

Thus, the trends discussed should take into account not only the formal ratio of the charges and their accessibility but also the relative stability of the complexes and their abilities to dissociate and partially leave the electrode interface in the presence of the low-molecular electrolytes of the buffer. To level the possible influence of electrolytes, HEPES buffer was used in the characterization of GCE/thiacalix[4]arene dendrimer/DNA sensors. 

**Figure 8:**
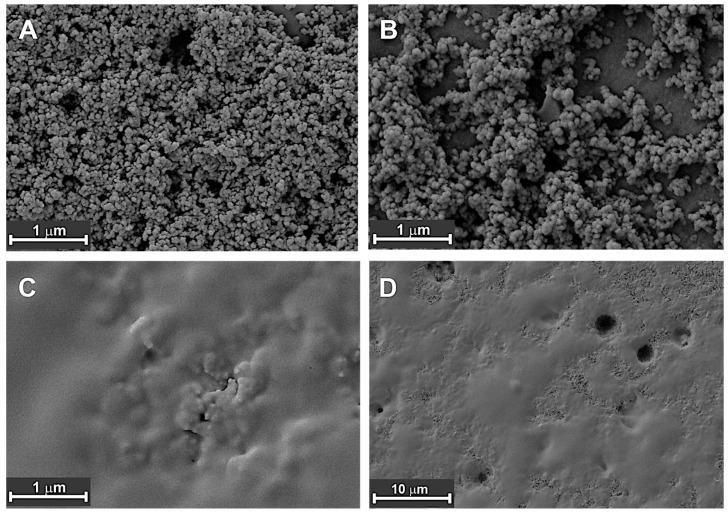
SEM images of the GCE covered with CB (**A**) and with macrocycle **2** (0.05 mM, aliquot 5 μL per electrode) (**B**) and DNA from salmon testes, 0.1 mg/mL (**C**,**D**).

**Figure 9:**
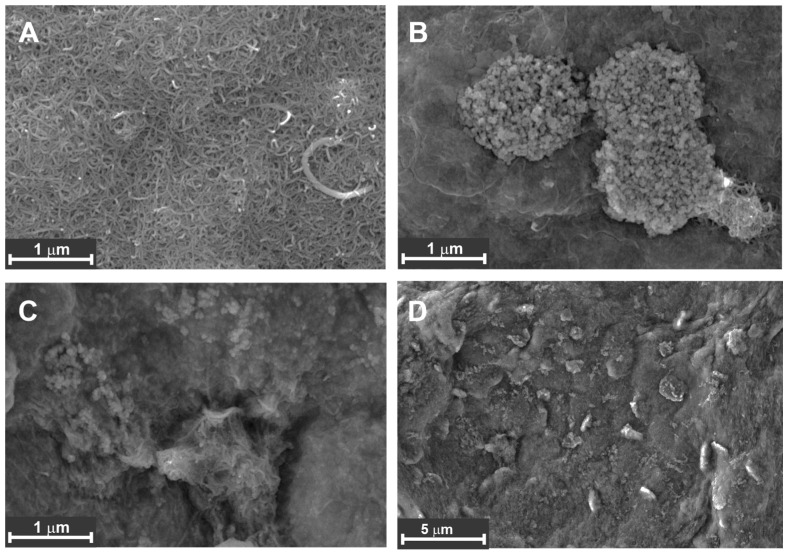
SEM images of the GCE covered with MWCNTs (**A**) and with macrocycle **3** (0.05 mM, aliquot 5 μL per electrode) (**B**) and DNA from salmon testes, 0.1 mg/mL (**C**,**D**).

SEM was applied to prove the assembling of the macrocyclic dendrimers and DNA on the carbon nanomaterials tested. Appropriate images are presented in Figure 8 and Figure 9.

Deposition of CB in chitosan as a film-forming matrix on bare GCE resulted in the formation of a porous layer with well-defined roundish particles of about 15–20 nm in diameter (Figure 8A).

**Figure 10:**
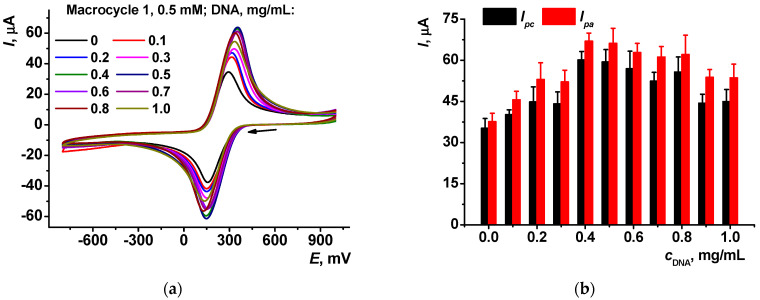

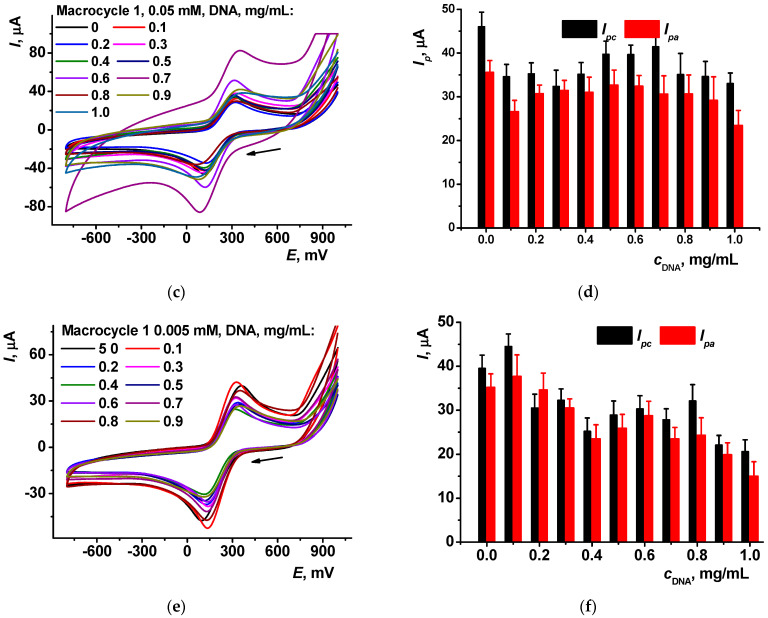
(**a**,**c**,**e**) Cyclic voltammograms of 5.0 mM K_3_[Fe(CN)_6_] recorded on the GCE covered with MWCNTs (2 μL of 1.3 mg/mL suspension in 0.375% chitosan), thiacalix[4]arene **1** (0.5, 0.05, and 0.005 mM in 2 μL aliquot per electrode) and various amounts of DNA from salmon tests. Arrow indicates the direction of the potential scanning. (**b**,**d**,**f**) The comparison of the reduction and oxidation peak currents. Average ± S.D. for four individual sensors.

Following application of the thiacalix[4]arene solution, the morphology of the layer was preserved, but the size of the particles increased by 15–20% due to adsorption of the macrocycle molecules (Figure 8B). Incubation of the electrode in the DNA solution resulted in a full change of the interface and formation of a thin film with randomly distributed defects, where the structure of underlying particles remained visible (Figure 8C,D). Moreover, rather big holes with the diameter of 0.8–1.2 µm appeared on the whole working area of the electrode. It could not be established if they penetrated through the layer to the bare glassy carbon or were limited by the carbon material layer. The reasons of their formation are not clear. One of the suggestions assumes the desorption of the complexes after neutralization of their charge in the self-aggregation of thiacalix[4]arene dendrimers and DNA molecules from the surface fragments with no CB particles (see Figure 10b,d for comparison).

MWCNTs being deposited on the GCE surface in the same chitosan matrix showed a typical worm-like structure with a high internal pore volume. 

Contrary to CB behavior, adsorption of the macrocycle resulted in full coverage of the particles with the formation of a solid film with a wavy surface and randomly distributed microcrystalline inclusions of micrometer size. They probably are formed by aggregated macrocycle molecules and disappear after contact with the DNA, promoting the formation of appropriate complexes via electrostatic interactions. Nevertheless, the heterogeneity of the layer was retained with ellipsoid particles placed onto the wrinkles of the film. The difference in the assembling of thiacalix[4]arene dendrimers in DNA on the CB and MWCNTs surface can be attributed to the different specific surface of the nanoparticles available for interactions and the more hydrophobic character of the MWCNT’s walls limiting the attraction of charged terminal groups of the macrocycles. DNA application obviously affects both the self-aggregation of the reaction partners and the charge distribution of the electrode.

### 3.3. EIS Characterization of the Surface Layer and DNA Target Interactions

#### 3.3.1. Detection of the DNA Damage

**Scheme 1:**
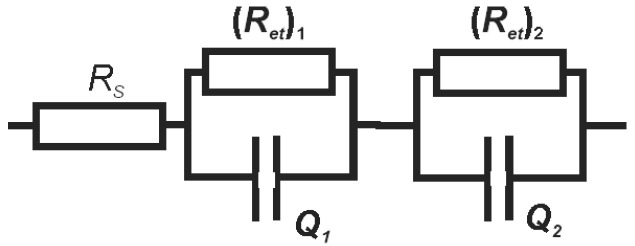
Equivalent circuit for the Nyquist diagram fitting.

EIS offers broad opportunities for the characterization of the surface film assembling and of the processes that involve DNA molecules adsorbed onto the thiacalixarene dendrimers. Any changes of the surface layer influencing the DNA–thiacalixarene interaction in terms of charge separation/shielding and accessibility of the negatively charged redox indicator (0.01 M [Fe(CN)_6_]^3−/4−^ equimolar mixture) would result in appropriate changes of the charge transfer resistance and surface layer capacitance. The equivalent circuit applied for the EIS data fitting is presented in Scheme 1. Here, *R_et_* is the electron transfer resistance and *Q* constant phase element and *R_s_* is the electrolyte resistance. Index 1 corresponds to the solution–modifier interface and index 2 to that of the electrode–modifier interface. EIS measurements were performed at equilibrium potential equal to 0.274 V. It should be noted that the exponential factor *N* at the constant phase element was higher than 0.99 for all the electrodes and measurement conditions, so that its behavior corresponded to the pure capacity of the electrode interface.

**Figure 11:**
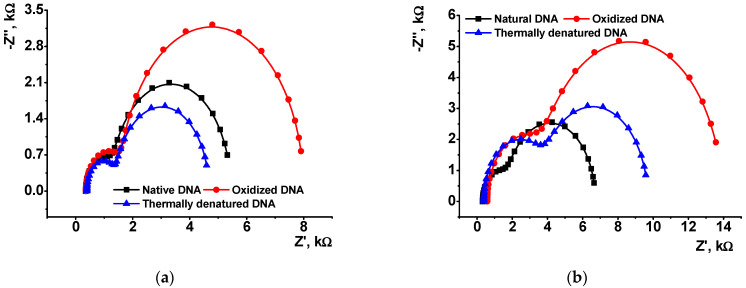
The Nyquist diagrams of the impedance spectra recorded on the GCE covered with thiacalixarene **2**, 0.05 mM per electrode, and DNA from salmon testes, 0.1 mg/mL aliquot 5 µL per electrode. Comparison of native, thermally, and chemically damaged DNA (**a**) at 10 min. incubation; (**b**) at 60 min. incubation. An amount of 0.01 M [Fe(CN)_6_]^3−/4−^, frequency range from 100 kHz to 0.04 Hz, amplitude of the applied sine potential 5 mV, 0.1 M HEPES, pH = 7.0.

As an example, Figure 11 shows the Nyquist diagrams obtained on the electrodes modified with the CB and thiacalix[4]arene **2**/DNA complex containing native, chemically, and thermally denatured DNA from salmon testes. The DNA treatment was equal to 10 (Figure 11a) and 60 min. (Figure 11b).

In these diagrams, semicircles corresponded to the electron exchange reactions on the internal (electrode–modifier) and external (modifier–electrolyte) interfaces. The EIS parameters are presented in Table 1. A low χ^2^ value proves the satisfactory quality of the fitting protocol.

Implementation of DNA generally increased the electron transfer resistance on the outer interface due to the implementation of nonconductive biopolymer molecules and the negative charge of the film. The effect even increased after the preliminary oxidation of DNA with the Cu^2+^/H_2_O_2_ mixture generated reactive oxygen species [41]. Meanwhile thermal denaturation of DNA slightly decreased the resistance, probably due to the higher flexibility of the single-stranded DNA formed and the better neutralization of the charge caused by phosphate residues of the DNA backbone with amino groups of the macrocycle substituents. After 10 min. incubation, changes on the electrode–modifier interface are rather small, and all the trends described above are related to the outer interface of the electrode. An increase in the incubation period to 60 min. resulted in a higher distinguishing of the DNA variously treated and extension of the changes in the resistance on the inner interface of the surface layer. This can be attributed to the distortion of the macrocyclic dendrimer layer and the loosening regularity of the surface layer content. 

**Figure 12:**
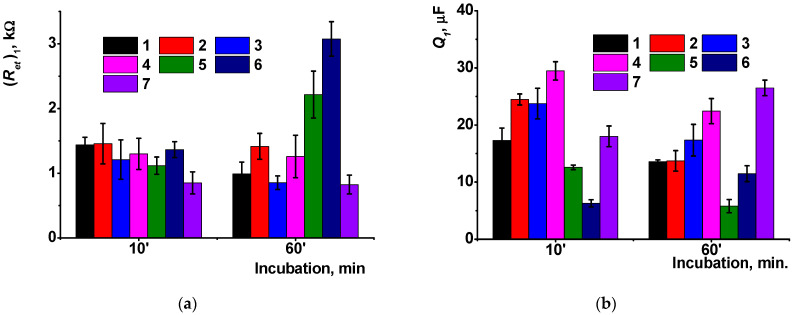

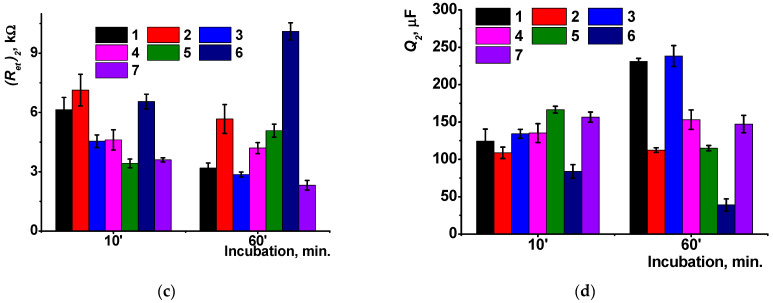
(**a**) EIS parameters measured on the GCE covered with CB and 0.05 mM thiacalix[4]arene **3** with native (1), thermally denatured (2), and chemically oxidized (3) DNA from salmon tests, native (4), thermally denatured (5), and chemically oxidized DNA from chicken erythrocytes (6) and PSS (7). (**a**) Charge transfer resistance and (**b**) capacitance of the electrode-modifier interface; (**c**) charge transfer resistance and (**d**) capacitance of the modifier–solution interface. See also description of the EIS parameters in Scheme 1; 0.01 M [Fe(CN)_6_]^3−/4−^, average ± S.D. for five electrodes.

Similar changes were observed for high-molecular DNA from chicken erythrocytes (Figure 12). For them, maximal changes were observed after 60 min. incubation in an oxidative mixture. Substitution of DNA with anionic polyelectrolyte poly(styrene sulfonate) (PSS) showed similar changes in the EIS parameters. This confirms the hypothesis about predominantly electrostatic interactions within the layer governing the electron exchange with the participation of the external, diffusionally free redox indicator.

Maximal changes in the EIS parameters after the DNA damage were observed for high-molecular DNA from chicken erythrocytes. This might result not only from the higher flexibility of the molecule caused by its unwinding or partial oxidation but also by formation of DNA fragments leaving the electrode interface. Slower changes in the DNA structure can also affect the relative changes of the appropriate parameters. 

The influence of PSS was the lowest one in the range of native and damaged DNA from various sources. This might result from the higher flexibility of the PSS molecules against native DNA so that the electrostatic interactions with macrocyclic counterparts were rather stable within the varied incubation period. 

Changes in the capacitance within the considered conditions of the DNA treatment were rather irregular. They are mostly dependent on the charge separation on the appropriate interface but not on changing the permeability of the surface layer for the redox indicator. Thus, they did not reflect the DNA damage as sensitively as the electron transfer resistance, depending both on diffusional and electrostatic factors. However, high absolute values of the capacitance indirectly confirmed the formation of charged complexes on the GCE surface.

#### 3.3.2. Doxorubicin Determination

Previously, we found that the thiacalixarene/DNA complex changed the signal of redox indicators after its incubation in doxorubicin solution as a model intercalator [38]. It was interesting to compare the characteristics of the drug determination using other configurations of the macrocyclic dendrimers in the DNA sensor assembly. 

**Figure 13:**
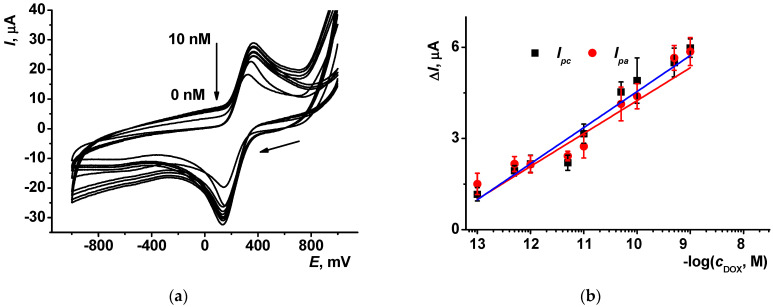
(**a**) Cyclic voltammograms recorded on the GCE covered MWCNTs, thiacalixarene **2** (0.05 mM per electrode), and DNA from salmon testes (0.1 mg/mL, aliquot 5 µL per electrode) in the presence of 0, 0.1, 0.5, 1.0, 5.0, 10, 50 pM, 0.1, 0.5, and 1.0 nM doxorubicin, incubation 10 min.; 0.1 M HEPES, pH = 7.0, 100 mV/s. Arrow indicates the direction of the potential scanning. (**b**) The dependence of the absolute current shift for anodic and cathodic peak potentials of [Fe(CN)_6_]^3−^ recorded in the above conditions on the doxorubicin concentration.

Here, the GCE with deposited MWCNTs was covered with 0.05 mM thiacalix[4]arenes **1**–**3** and 0.4 mg/mL DNA and incubated for 10 min. in the doxorubicin solution followed by measurement of cyclic voltammograms in the 0.5 mM [Fe(CN)_6_]^3−^ solution. The conditions for the doxorubicin measurement were established for the CB-covered GCE earlier. The use of hydroquinone as a redox indicator did not show significant changes in the recorded peaks. The Methylene green tested earlier changed the peak currents irregularly. Only the ferricyanide ions consecutively increased the oxidation and reduction peak currents when the doxorubicin concentration ranged from 0.1 pM to 1.0 mM (Figure 13).

Appropriate calibration curves are linearized in the semilogarithmic plots as follows (Equations (1) and (2)):

Δ*I_pa_*, μA = (16.4 ± 0.1) + (1.18 ± 0.11) log(*c*_DOX_, M), *R*^2^ = 0.994, n = 9 (1)

Δ*I_pc_*, μA = (15.1 ± 1.1) + (1.08 ± 0.15) log(*c*_DOX_, M), *R*^2^ = 0.947, n = 9 (2)

We could see that the deviation of the signal and linearization of the calibration plot allow semiquantitative determination of the doxorubicin in real samples but high sensitivity partially compensates for this drawback of the DNA sensor developed.

At a higher doxorubicin concentration, the direction of the peak current changed in reverse direction and the decay of the current was observed for the doxorubicin concentration higher than 10 nM. Such a behavior of the DNA sensor is quite opposite to that observed for other DNA sensors based on electrostatically accumulated biopolymer molecules when the intercalation resulted in a decrease in the currents with no respect for the redox indicator nature. At lower doxorubicin concentrations, the deviation of the signal becomes higher than 15% and prevents the quantification of the analyte content.

We can suppose that MWCNT-based biosensors either sense the doxorubicin by disaggregation of the DNA complexes or react on the higher hydrophobicity of the loading due to rather high content on the support with polyaromatic walls of the nanotubes. Testing changes in the configuration of thiacalixarenes resulted in a remarkable decrease in the slope of the curves to 0.8 μA/pC for *partial cone* (macrocycle **2**) and 0.65 μA/pC for *1,3-alternate* (macrocycle **3**). In both cases, reversed changes of the peaks were observed starting from 1 nM of doxorubicin. In all the DNA sensors, the anodic and cathodic peak currents changed synchronously so that their symmetry on cyclic voltammograms were retained in the whole range of the concentrations studied. Thermal denaturation of DNA as well as substitution of DNA with PSS dramatically suppressed the sensitivity toward doxorubicin down to its full disappearance. Thus, the limit of detection (LOD) calculated from S/N = 3 ratio was found to be 1.0 pM for the macrocycle **1**, 4.5 pM for the macrocycle **2,** and 10 pM for the macrocycle **3**, whereas the same thiacalix[4]arenes with thermally denatured DNA showed the LODs of 50, 100, and 500 pM, respectively. All the DNA sensors were used only once without any treatment because preliminary experiments showed irregular changes of the redox indicator signals in the following contacts of the DNA sensor with intercalator. Although the content of the DNA sensor layer was not optimized for drug determination and the study of surface layer assembling was the primary goal of the work, the sensitivity of doxorubicin detection was quite comparable with other DNA sensors described in the literature. The comparison of the electrochemical DNA sensors for doxorubicin determination is presented in Table 2. 

Lower LODs were reported for the biosensors based on thin layers of redox active polymers (poly(Azure A), copolymers of Methylene blue and Neutral red) and physically adsorbed Acridine Yellow. In these sensors, DNA was placed in direct physical contact with the mediator system and effectively altered the redox equilibria on the electrode.

#### 3.3.3. Stability, Selectivity, Reproducibility, and Real Sample Analysis

The signal toward doxorubicin was quite stable within two weeks of storage in working buffer at 4 °C. The assessment of the relative changes in the doxorubicin response prolonged the working period for one month. The sensor-to-sensor repeatability was assessed using five individual sensors and 50 pM of doxorubicin solution. S.D. was found to be 7.2% for freshly prepared sensors and about 12% for those after two weeks’ storage. Average concentration determined did not drift within the testing period.

Changes in the ferricyanide peaks on cyclic voltammograms were selective toward doxorubicin and did not alter in the presence of common compounds used as stabilizers of medications (mannitol, hydroquinone, glucose, lactose). The use of spiked samples of normalized human serum rehydrated in phosphate buffer saline, pH 7.4, showed the recovery of doxorubicin of 120% for 0.1 nM and 105% for 1.0 nM doxorubicin. The addition of bovine serum albumin (41.4 mg/mL) did not affect the influence of doxorubicin on the ferricyanide signals.

Other anthracycline preparations affected the signal of the ferricyanide redox indicator similarly to doxorubicin. This coincides well with the general mechanism of their interaction with DNA molecules. All the medications tested (daunorubicin, idarubicin) have the same anthracycline core and differ from each other by small substituents that changed the hydrophilicity of the molecule but not the intercalation mechanism of their interaction with DNA helix. Interference with the doxorubicin was assessed using the IC_30_ (analyte concentration exerting 30% shift of the signal). It was equal to 5 pM for doxorubicin, 40 pM for daunorubicin, and 60 pM for idarubicin (DNA sensor based on the macrocycle **1**). This coincides well with direct comparison of the response to these anthracycline medications performed with a polyaniline–DNA sensor [42]. The LODs of 0.01 nM doxorubicin, 0.1 nM daunorubicin, and 0.2 nM idarubicin were obtained using the EIS technique. It should be noted that chemotherapy assumes a mostly separate usage of anthracycline drugs. Sulfonamide preparations (sulfamethoxazole) were shown to be rather inert, both alone and in the mixture with doxorubicin [42]. In our study, IC_30_ was equal to 0.01 mM for sulfamethoxazole dissolved in normalized human serum.

As a real sample assay example, medications (Doxorubicin-LANS ^®^, mannitol in 4:1 mass. ratio) and Doxorubicin—TEVA ^®^ (lactose in 5:1 mass. ratio) were tested. Both preparations were dissolved in deionized water, and then, the doxorubicin content was determined using calibration plot obtained with standard solutions. The recovery was found to be 95–110% depending on the nominal concentration of the drug (10 and 100 pM).

## 4. Discussion

The influence of various factors on the assembling of complexes consisting of dendrimers of thiacalix[4]arene core and DNA has been considered to assess the principal factors that determine the electrochemical performance of appropriate DNA sensors. It was shown that the configuration of thiacalix[4]arene derivatives bearing eight terminal amino groups in the substituents of the lower rim affected the permeability of the surface layer for diffusionally free redox indicators (ferricyanide ions) due to the electrostatic interactions and self-aggregation ability that changed the charge and the size of the aggregates on the electrode interface. The ratio of the macrocycles and DNA quantities deposited on the electrode affected the signals of the redox indicators differently depending on their charge (negatively charged ferricyanide, neutral hydroquinone, and positively charged Methylene green) and the density of the surface layer. Contrary to the *cone* configuration of the macrocycle, *1.3-alternate* and *partial cone* could change the charge of the complex with no regard for the saturation of the DNA molecule due to opposite position of the amino groups against the plane of the macrocyclic core (see Figure 6 for possible assembling of appropriate complexes). Such reactions were also proved previously by UV-Vis spectrophotometry and dynamic light scattering [37]. The DNA sensors assembled on the MWCNTs’ support showed behavior different from that on the CB underlying layer because of the different morphology of the surface layer with implementation of self-aggregates of the macrocycles able to further disaggregation after contact with the DNA. The DNA sensors were tested in the discrimination of native, thermally, and chemically damaged DNA and in determination of doxorubicin as the model intercalator with the LOD of 1.0 pM (semilogarithmic scale) and recovery of 105–120% (spiked normalized human serum, Doxorubicin-TEVA^®^ and Doxorubicin-Lans^®^). Thus, the role of macrocycle configuration, charge of redox indicator, and the supporting carbon material has been explored, and the importance of electrostatic interactions and aggregation of the reactants on the DNA accessibility for small molecules (doxorubicin) and redox indicators was shown. The results can be applied in the design of drug delivery systems. After further optimization of the assembling directed towards the stabilization of the signal, the developed DNA sensors can find application in preliminary screening of antitumor drugs and thermal damage of DNA. They can also be applied for testing potential drug/DNA nanocontainers as future delivery systems.

## Figures and Tables

**Table 1:** EIS parameters obtained for the GCE covered with CB and macrocycle 2 (*partial cone*) with natural, thermally denatured, and chemically oxidized DNA.

DNA Coating	*R_s_*, Ω	Electrode–Modifier Interface			Modifier–Solution Interface			
		*R*_2_, Ω	*Q*_2_, μF	*N*_2_	*R*_1_, Ω	*Q*_1_, μF	*N*_1_	χ^2^
Native DNA, 10 min.	344 ± 21	1300 ± 120	30 ± 6	0.9995	4612 ± 2100	135 ± 224	0.9998	0.007
Native DNA, 60 min.	328 ± 21	1260 ± 126	22 ± 5	0.9993	4200 ± 270	153 ± 53	0.9997	0.013
Denatured DNA. 10 min.	344 ± 16	1117 ± 104	13 ± 5	0.9998	3420 ± 212	166 ± 44	0.9998	0.008
Denatured DNA. 60 min.	360 ± 18	2215 ± 80	6± 1	0.9994	5080 ± 280	115 ± 16	0.9996	0.008
Oxidized DNA, 10 min.	353 ± 30	1365 ± 122	6 ± 1	0.9992	6550 ± 570	84 ± 10	0.9996	0.001
Oxidized DNA, 60 min.	340 ± 25	3076 ± 166	11 ± 2	0.9996	10105 ± 432	79 ± 18	0.9998	0.007

**Table 2:** Comparison of analytical characteristics of doxorubicin determination with electrochemical DNA sensors.

Modifier	Signal Measurement Mode	LOD	Ref.
Polyaniline	CV, [Fe(CN)_6_]^3−^ redox indicator	10 pM	[42]
Poly(Neutral red)	CV (Neutral red), EIS ([Fe(CN)_6_]^3−^ redox indicator)	0.05 nM (Neutral red), 0.1 nM (EIS)	[43]
Poly(Azure B)	CV (poly(Azure B)	70 pM	[44]
Poly(Azure A)	CV (poly(Azure A)	0.03–0.3 pM	[45]
Pillar[5]arene—poly(Methylene blue)—poly(Neutral red)	CV (Neutral red)	0.13 pM	[46]
Poly(proflavine)	EIS ([Fe(CN)_6_]^3−^ redox indicator)	0.3 nM	[47]
Single-walled carbon nanotubes	DPV (guanine oxidation in DNA molecules)	0.6 nM	[48]
Pt and Ag nanoparticles	DPV (guanine oxidation in DNA molecules)	0.1 ppm	[49]
Acridine Yellow	CV	0.07 pM	[50]
Poly(Azure B—proflavine)	EIS ([Fe(CN)_6_]^3−^ redox indicator)	0.01 nM	[51]
Macrocyclic dendrimers on thiacalix[4]arene core	CV, [Fe(CN)_6_]^3−^ redox indicator	1–10 pM	This work

CV—cyclic voltammetry, EIS—electrochemical impedance spectroscopy, DPV—differential pulse voltammetry.

## Data Availability

Data is contained within the article.
